# Landscape of PCOS co-expression gene and its role in predicting prognosis and assisting immunotherapy in endometrial cancer

**DOI:** 10.1186/s13048-023-01201-6

**Published:** 2023-07-01

**Authors:** Yun Zhang, Yifang Hu, Jian Yu, Xiaoyan Xie, Feng Jiang, Chuyan Wu

**Affiliations:** 1 Department of Rehabilitation Medicine, The First Affiliated Hospital of Nanjing Medical University, No.300, Guangzhou Road, Nanjing, 210029 China; 2 Department of Geriatric Endocrinology, The First Affiliated Hospital of Nanjing Medical University, Nanjing, China; 3 Department of Neonatology, Obstetrics and Gynecology Hospital of Fudan University, No.419, Fangxie Road, Shanghai, 200011 China

**Keywords:** Endometrial cancer, Polycystic ovarian syndrome, Steroid hormone biosynthetic process, Prognosis, Immunotherapies

## Abstract

**Background:**

Endometrial carcinoma (EC) is the sixth most frequent malignancy in women and is often linked to high estrogen exposure. Polycystic ovarian syndrome (PCOS) is a known risk factor for EC, but the underlying mechanisms remain unclear.

**Methods:**

We investigated shared gene signals and potential biological pathways to identify effective therapy options for PCOS- and EC-related malignancies. Weighted gene expression network analysis (WGCNA) was used to identify genes associated with PCOS and EC using gene expression data from the Gene Expression Omnibus (GEO) and Cancer Genome Atlas (TCGA) datasets. Enrichment analysis using Cluego software revealed that the steroid hormone biosynthetic process was a critical feature in both PCOS and EC. A predictive signature encompassing genes involved in steroid hormone production was developed using multivariate and least absolute shrinkage and selection operator (LASSO) regression analysis to predict the prognosis of EC. Then, we conducted further experimental verification.

**Results:**

Patients in the TCGA cohort with high predictive scores had poorer outcomes than those with low scores. We also investigated the relationship between tumor microenvironment (TME) features and predictive risk rating and found that patients with low-risk scores had higher levels of inflammatory and inhibitory immune cells. Also, we found that immunotherapy against anti-CTLA4 and anti-PD-1/PD-L1 was successful in treating individuals with low risk. Low-risk individuals were more responsive to crizotinib therapy, according to further research performed using the “pRRophetic” R package. We further confirmed that IGF2 expression was associated with tumor cell migration, proliferation, and invasion in EC cells.

**Conclutions:**

By uncovering the pathways and genes linking PCOS and EC, our findings may provide new therapeutic strategies for patients with PCOS-related EC.

**Supplementary Information:**

The online version contains supplementary material available at 10.1186/s13048-023-01201-6.

## Introduction

According to the diagnostic criteria employed, Polycystic Ovary Syndrome (PCOS), the most prevalent endocrine condition, affects between 6% and 20% of women of reproductive age [1, 2]. While the PCOS are not yet fully understood, an increasing body of evidence suggests that it may be a complex polygenic disease affected by environmental and lifestyle factors. Women of reproductive age with PCOS may experience a range of health issues, including obesity, insulin resistance, metabolic disorders, infertility, heart disease, and cancer, in addition to the hyperandrogenemia and anovulation symptoms commonly associated with the condition [3]. Thanks to recent advancements in genetic tools and procedures, large-scale genetic testing has been used to explore the genetic underpinnings of PCOS. Numerous studies have investigated candidate genes associated with PCOS in an effort to better understand the condition’s genetic correlations [4].

The incidence and death rates of endometrial carcinoma (EC), the sixth most prevalent malignancy in women, are rising. It is estimated that by 2030, the prevalence of this disease will increase by 50–100% [5, 6]. Endometrial cancer is commonly associated with excessive estrogenic stimulation of the endometrium, leading to mitotic stimulation and eventual malignant transformation of the endometrial glandular epithelium. This hormone-sensitive disease is well-known for its reliance on estrogen and its effects on the endometrium [7]. Several risk factors have been identified for EC, including obesity, miscarriage, PCOS, type 2 diabetes, insulin resistance, and estrogen exposure therapy. These factors have been associated with an increased likelihood of developing EC and should be considered when assessing an individual’s risk of developing the disease [8].

In contrast to control groups, several meta-analyses have shown that women with polycystic ovarian syndrome (PCOS) had a much greater prevalence of EC, with a three- to four-fold increase. These findings suggest a strong association between PCOS and an increased risk of developing EC, underscoring the importance of close monitoring and preventive measures for women with PCOS [9, 10]. Furthermore, certain studies have suggested that PCOS may have an adverse effect on the prognosis of EC. This highlights the need for continued research into the relationship between these conditions and the development and management of effective treatment strategies [11].

**Fig. 1:**
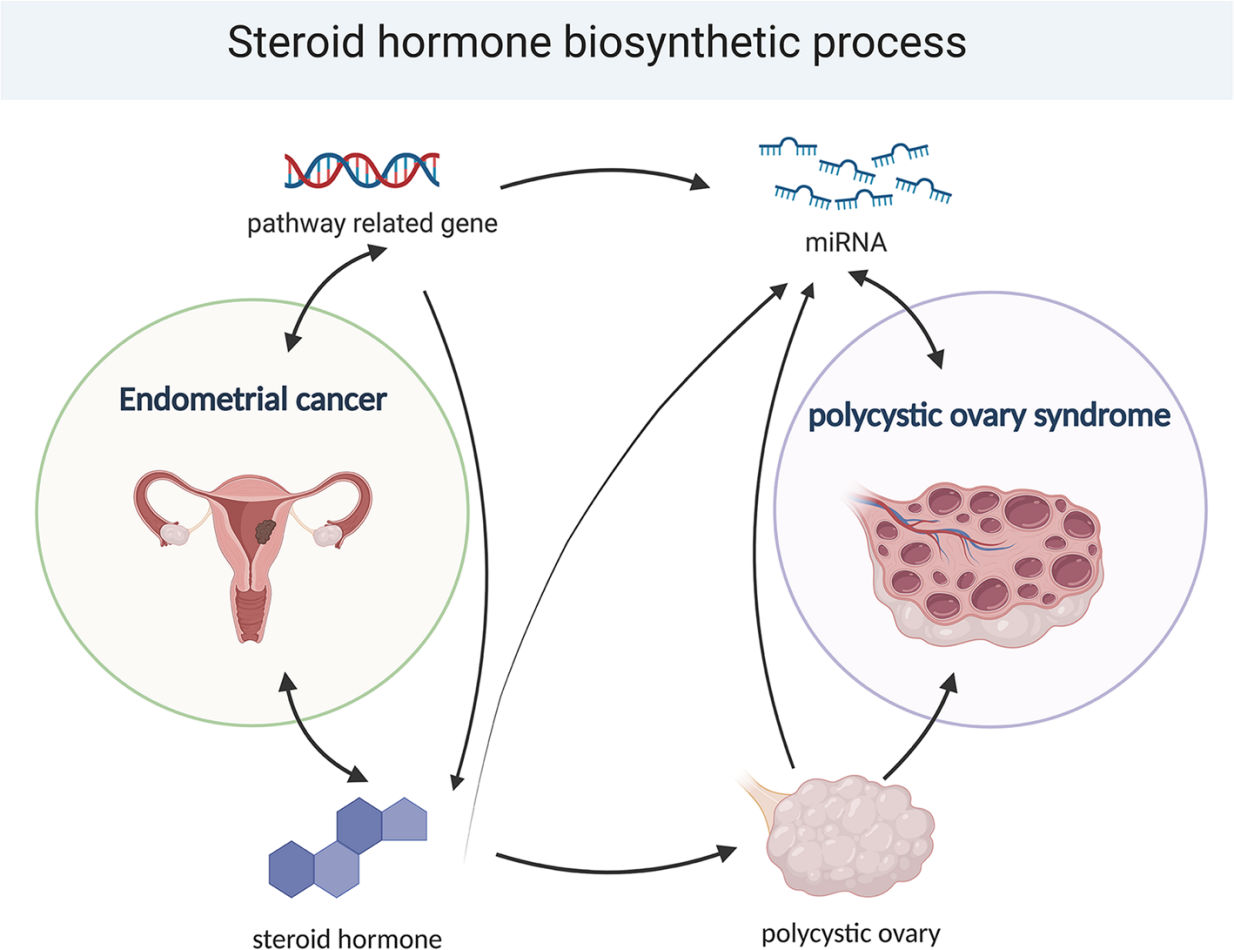
Abstract graph

**Fig. 2:**
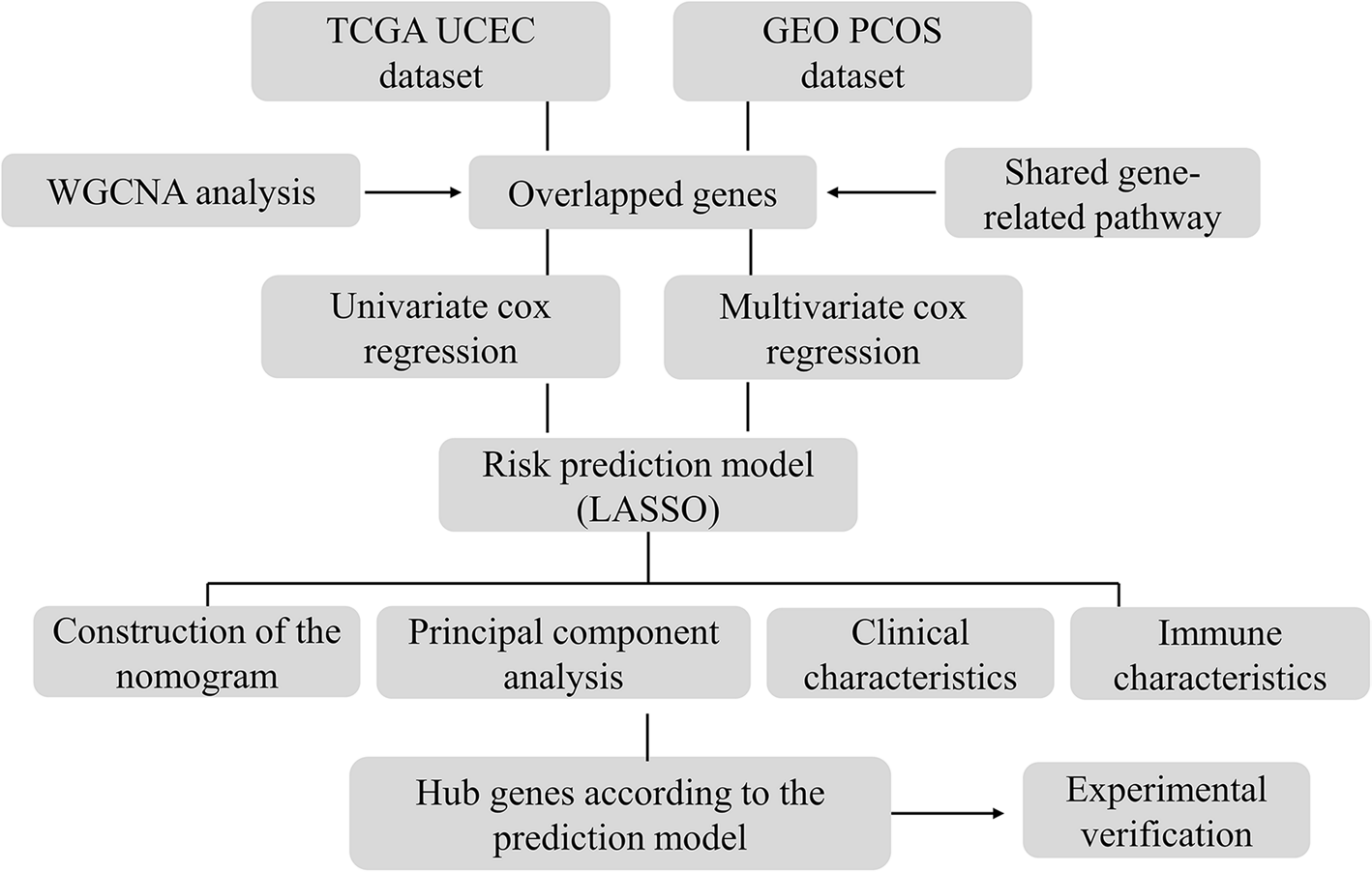
Flow chart

This study’s goal was to investigate possible PCOS and EC connection mechanisms. By utilizing weighted gene co-expression networks, researchers were able to identify genes that are shared between PCOS and EC, as well as potential biochemical pathways involved in their interaction. This method may open the door to the creation of fresh therapies and preventative measures by revealing important insights into the processes underlying the connection between these two illnesses (Fig. 1 Abstract graph and Fig. 2 Flow chart).

## Methods

### Downloading and analyzing data

To identify PCOS gene expression profiles, we conducted a thorough search of the GEO database using the specific keywords. The dataset underwent several filtration processes to ensure its accuracy and relevance. Firstly, the gene expression profiling of both patients and controls was included. Secondly, we selected uterus tissue as the preferred sequencing arrangement. Thirdly, we ensured that each group’s sample size was not less than 10 to guarantee accurate results from the Weighted Gene Co-expression Network Analysis (WGCNA). Lastly, the data files must contain either processed or raw data that can be reanalyzed.

We conducted a search for PCOS gene expression profiles in the GEO database using the specific keywords, and ultimately chose GSE43322 as the relevant dataset for our analysis. The authors of this dataset provided several matrix files containing normalized data processed by the mas5 algorithm. To prepare the transcriptomic profile for analysis, we converted it to log2 format and matched the probes to their corresponding gene symbols using the platform’s annotation document. Finally, we created a gene matrix with the sample name serving as the row name and the gene symbol as the column name. In addition, we retrieved raw RNA sequencing (RNA-seq) data sets for Uterine Corpus Endometrial Carcinoma (UCEC) samples from the TCGA database, along with clinical data such as age, BMI, grade, and predictive variables. To prepare the UCEC datasets for analysis, we translated the Ensembl Gene IDs into their corresponding gene symbols using the annotation platform. For cases where multiple probes were directed at the same Entrez Gene ID, we applied the average value. Finally, we performed a log2 (FPKM + 1) transformation on the TCGA datasets.

### Weighted gene Co-Expression network analysis

The WGCNA is a method that identifies coexpression gene modules with significant biological relevance and evaluates the association between gene networks related to diseases [12]. We employed WGCNA to identify modules related to PCOS and UCEC. Using the “WGCNA” tool in R.4.0.4 software, we conducted WGCNA analysis. The appropriate power value was chosen through the “pick soft threshold” tool in the “WGCNA” package, following the scale-free networking system within the range of 1 to 20. Once we had determined the optimal power value, we built the proximity matrix and matched the gene distribution to the scale-free network based on the degree of connectivity. The topological matrix (TOM matrix) was then calculated, and the genes were re-clustered using the TOM matrix obtained from gene expression. In the end, we computed the correlation coefficient (cor) and its corresponding p-value (P value) to establish the association between various gene modules and clinical traits.

### Enrichment analysis of shared genes

We selected courses that were most relevant to PCOS and UCEC. Next, we used Jvenn to compare the shared genes in categories that showed an adverse relationship with PCOS and UCEC [13]. The plug-in ClueGO in Cytoscape can be utilized to create a network of interconnected sentences that categorize non-redundant GO terms [14]. ClueGO conducted a scientific inquiry to look at the probable relevance of these common genes in PCOS and UCEC. A p-value of 0.05 or below was utilized to define significance for the gene ontology (GO) assessment, which was primarily focused on the biological process.

### Building risk prediction models gene model linked to the synthesis of steroid hormones

We investigated the genes related to the steroid hormone biological process by querying GO and KEGG pathway-related genes (GO0120178 and hsa00140), removing any duplicates, and obtaining a total of 78 genes (Supplement File1). We then compared the prognosis results for each sample with the expression levels of differentially expressed genes (DEGs) associated with UCEC. To select the genes associated with prognosis, we employed univariate and multivariate Cox regression techniques on the DEGs from the TCGA cohort and chose genes with a P value of less than 0.001. The “maftools” R software package was used to examine the mutation and gene interactions in cancer tissues from the training dataset. Finally, we used the “glmnet” R software package to further analyze the prognosis-related genes and used LASSO to construct a forecast risk score model for UCEC. Ten cross-verifications were conducted to establish the penalty parameter (λ) of the model. Following is the process used to calculate the risk score for each sample.

$$\mathrm{Risk}\;\mathrm{Score}=\sum\nolimits_1^{\mathrm i}\mathit{(Coefi\;\ast\;ExpGenei)}$$

We denoted the transcriptome value obtained from the independent prognostic score model as “ExpGene,“ and the non-zero regression coefficient computed by LASSO as “Coef.“ We separated the samples into high-risk and low-risk subgroups based on the average value of the risk evaluations. The differences in overall survival (OS) between the subgroups with low and high scores were then compared using the Kaplan-Meier (K-M) and log-rank techniques. The ROC curve was created and the predicted risk score model’s accuracy rate was assessed using the R software package “survivalROC”. Using the test data, we finally verified the reliability of the risk-based scoring model.

### Analysis by principal components of the risk score model

To gain a more comprehensive insight into the notable distinctions between the two groups, we employed the limma software program to perform principal component analysis (PCA) on gene expression. Initially, we applied PCA to scrutinize the patterns of all differentially expressed genes (DEGs) associated with EC. The gene expression in the expected risk rating model was then investigated using PCA. Next, we used the “ggplot2” software application to plot the PCA findings in two dimensions.

### The relationship between clinical features and risk ratings

Using the sample ID as the connecting element, we combined each sample’s value with the pertinent clinical data. To explore the correlation between risk scores and clinical information, we employed the Limma R program. The dataset was partitioned into two groups according to clinical traits, and we utilized the Kruskal Wallis (K-W) method to compare the variations in risk scores between these subsets. For statistical significance to be established, a p value of 0.05 or less was used as the threshold.

### Comparison of Immune features between subgroups

To assess the dissimilarities in biological processes between the two groups, we employed the “gsva” R tool. We computed the IC50 of 5-Fluorouracil for each sample with the aid of the pRRophetic R program. The IC50 value is indicative of the drug’s potential to suppress particular biological or metabolic activities. Moreover, we determined the degree of immune-related infiltration in each patient in the TCGA dataset by employing ssGSEA and the “GSVA” and “GSEABase” R packages [15]. The enrichment index of the ssGSEA algorithm indicated the expression level of each immune-related feature, and we analyzed the differences in enrichment values between the two categories. Moreover, the association between immune cells and genes related to prognosis was also investigated. We used TIDE to predict the response to immune checkpoint inhibitors of CTLA-4 and PD-1 in the two risk score categories [16].

### Nomogram construction

Using the TCGA cohort, we constructed a nomogram to predict the overall survival (OS) of EC patients based on age, stage, grade, BMI, and an independent prognostic score model. The “RMS” software package in R was used to build the nomogram, and we developed a time-dependent calibration graph to assess its accuracy. We also conducted a multivariate Cox regression analysis to determine whether the prediction model could be used as a standalone marker of survival in EC patients. Additionally, we utilized a ROC curve to calculate the AUC and validate the nomogram’s predictive ability.

### Analysis of hub genes related to survival using the prediction model

Initially, we identified the differentially expressed genes (DEGs) with an adjusted p-value of 0.05 by comparing data from two subgroups using the limma R software package. We established a protein-protein interaction (PPI) network using the string database (https://string-db.org; version: 11.0) with interaction scores above 0.40 (median confidence). We subsequently employed the Cytoscape software (version 3.9.1) to further examine and visualize the PPI network data. Using a topological method implemented by the Cytoscape plug-in Cytohubba, we identified the central gene among all DEGs. Finally, one gene from the hub gene was chosen for model assessment, and we partitioned all samples into subgroups based on this gene’s expression. We conducted Kaplan Meier analysis to determine whether there was a difference in survival between the two groups. Furthermore, we investigated the presence of immune cells associated with this gene.“

### Cell culture and transfection

We cultured cells from different strains of EC (HEC-1 A, RL95-2, Ishikawa, KLE, AN3CA), as well as normal control cells hESC, in Dulbecco’s modified Eagle’s medium (DMEM) supplemented with 10% fetal bovine serum (FBS) at 37 °C in 5% CO2. Various functional studies were performed using logarithmic phase cells. To knock down or overexpress IGF2, EC cells were transfected with plasmid siIGF2 or IGF2-cDNA using Lipofectamine® 3000 according to the manufacturer’s instructions. The target sequence for siIGF2 was 5’-CGGCCTGGGAAGTAGGACTAA-3’.

### qRT-PCR

We extracted total RNA from the cells using Trizol reagent (Invitrogen, America) and generated cDNA with the HiScript Synthesis Kit (Vazyme, China). The PCR mixture was then prepared with cDNA, ddH2O, primers, and SYBR Green Master Mix. Finally, the qRT-PCR amplification was measured using the StepOnePlus Real-Time PCR system (Applied Biosystems, US). One of the primer sequences used was as follows: IGF2, forward-5’-GTGGCATCGTTGAGGAGTG-3’, reverse-5’-CACGTCCCTCTCGGACTTG-3’.

### CCK-8 and clone assay

To assess the proliferative capacity of EC cells, we conducted CCK-8 and plate colony tests. Cell growth was measured using the CCK8 kit (Beyotime, China) at intervals of 0 h, 24 h, 48 h, 72 h, and 96 h according to the manufacturer’s protocol. Enzyme labeling was used to measure 450 nm absorbance (Thermo, USA). For the colony test, approximately 500 cells from various groups were seeded into each well of a six-well plate. Once colonies formed, cells were fixed and stained using 4% paraformaldehyde and crystal violet.

### Transwell invasion assay

We transfected EC cells with si-IGF2 or IGF2-cDNA and seeded 1 × 105 cells in the upper chambers of Transwell, which were pre-coated with Matrigel (Biosciences, USA), in an empty DMEM medium. The lower chamber was filled with DMEM containing 10% FBS. After 24 h of incubation, we preserved the penetrated cells with 4% methanol and stained them with 0.1% crystal violet. We visualized and counted the cells under an inverted microscope (Nikon, Japan).

### Statistical analysis

We used the Wilcoxon rank sum test to investigate the differences between subgroups with low and high score. To ascertain the variations in overall survival (OS), we used Kaplan Meier analysis. To find the independent variables connected to OS in EC, we used Cox regression analysis. We used a ROC curve to assess the prognostic efficacy of the nomogram, clinical factors, and independent prognostic score model. R version 4.0.4 was used for all statistical calculations, and a significance threshold of P 0.05 was used.

## Results

### The co-expression modules in PCOS and EC and the common gene signatures in PCOS and EC

**Fig. 3:**
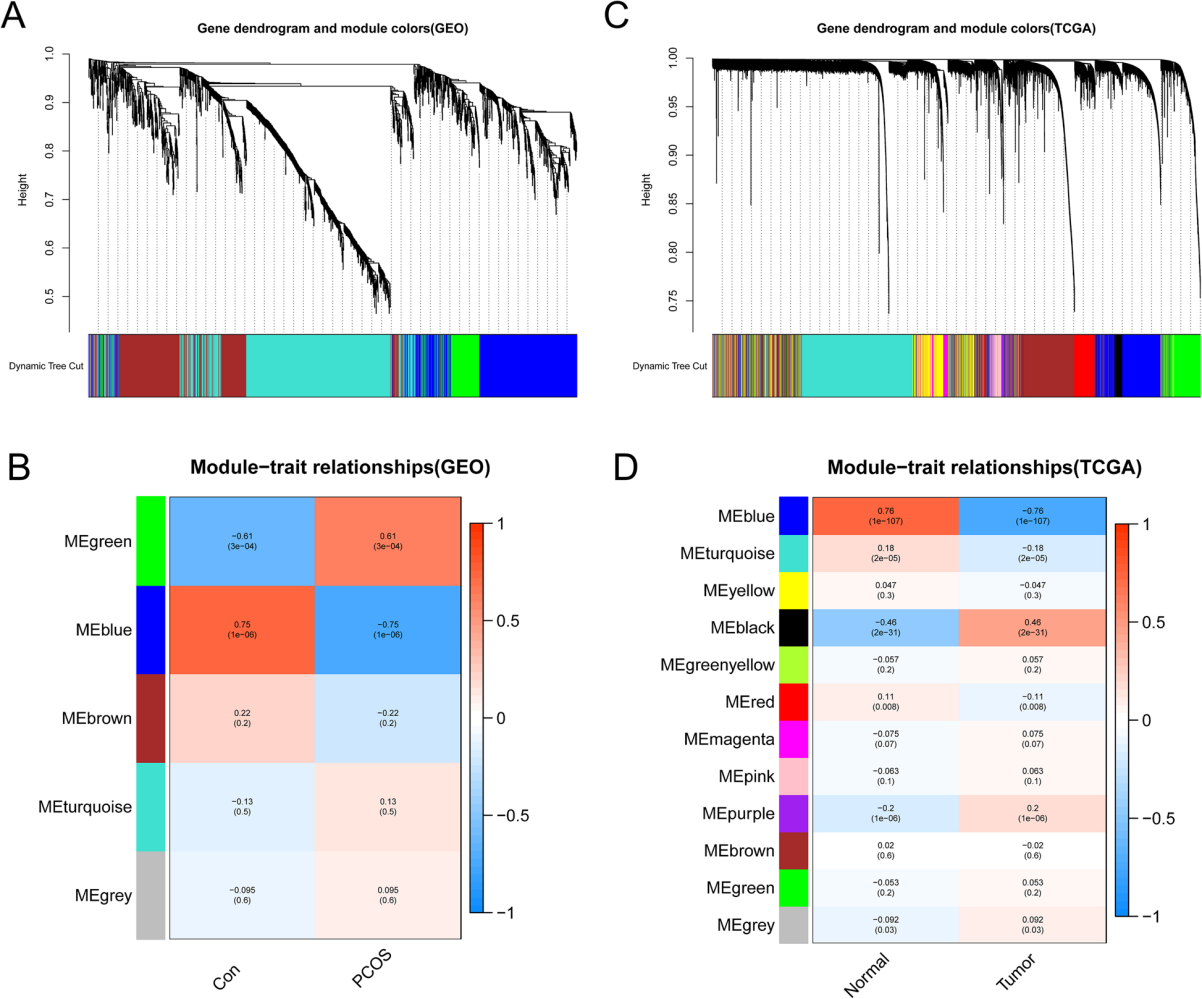
Weighted analysis of the network of gene co-expression (WGCNA). **A** The co-expression gene cluster dendrogram in PCOS. **B** Relationships between modules and traits in PCOS. The matching correlation and p-value are provided in each cell. **C** The co-expression gene cluster dendrogram in EC. **D** Relations between modules and traits in EC. The matching correlation and p-value are provided in each cell

Using WGCNA, we identified a total of 5 modules in the PCOS-GEO datasets, with each module denoted by a distinct hue. We created a heatmap using the Spearman correlation coefficient to assess the correlation between each module and the illness, with two modules, “green” and “blue,“ being chosen as PCOS-related due to their strong links with the condition (green module: *r* = 0.61, *p* = 3e-04; blue module: *r* = -0.75, *p* = 1e-06) (Fig. 3A and B). The green module contained 162 genes with a reasonable correlation with PCOS, while the blue module contained 553 genes with a negative correlation with PCOS. Similarly, we identified a total of 12 modules in the UCEC-TCGA datasets, with the “blue” module containing 3011 genes and having a very poor correlation with EC (*r* = -0.76, *p* = 1e-107). We visualized the modules and their correlation with the disease using a heatmap, as shown in Fig. 3 C and D.

**Fig. 4:**
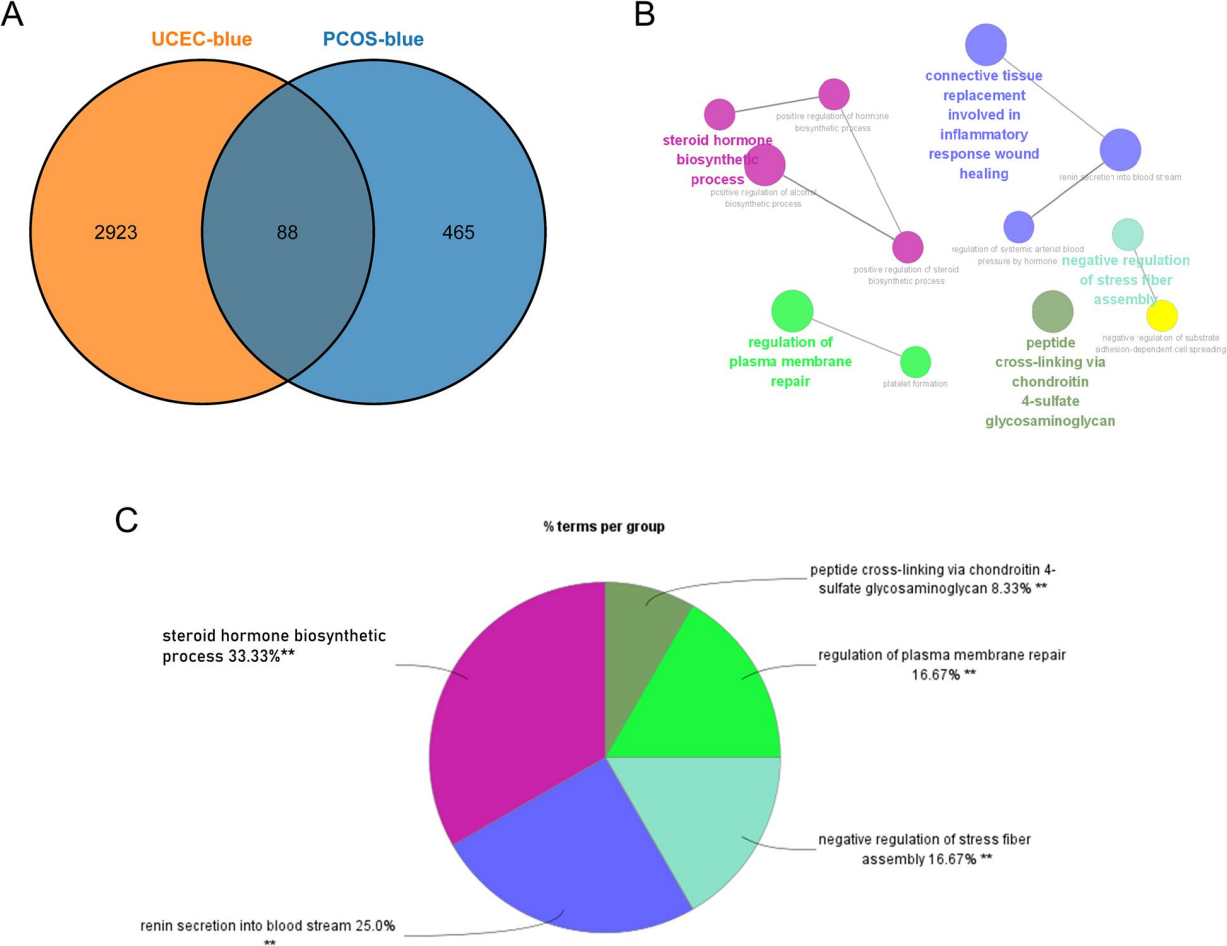
Enrichment analysis for ClueGO. **A** By overlaying them, the common genes between the blue modules of PCOS and EC. **B** The GO term interaction network produced by the ClueGO Cytoscape plug-in. Each group’s most useful phrase is highlighted. **C**The share of each GO word group in the total. GO, gene ontology, **p < 0.05

A set of 88 genes, which were negatively correlated with both PCOS and EC, were identified and labeled as gene set 1 (GS1), indicating their potential involvement in the pathophysiology of both conditions (Fig. 4A). To investigate the possible functions of GS1, GO enrichment analysis was performed using the GlueGo tool. The results showed that the top two significantly enriched GO terms were “renin secretion into the blood stream” and “steroid hormone biosynthetic process.“ The latter term accounted for 33.33% of all GO terms, suggesting that steroid hormone biosynthesis may play a crucial role in both PCOS and EC (Fig. 4B, C).

### Establishment of the prognostic model

**Fig. 5:**
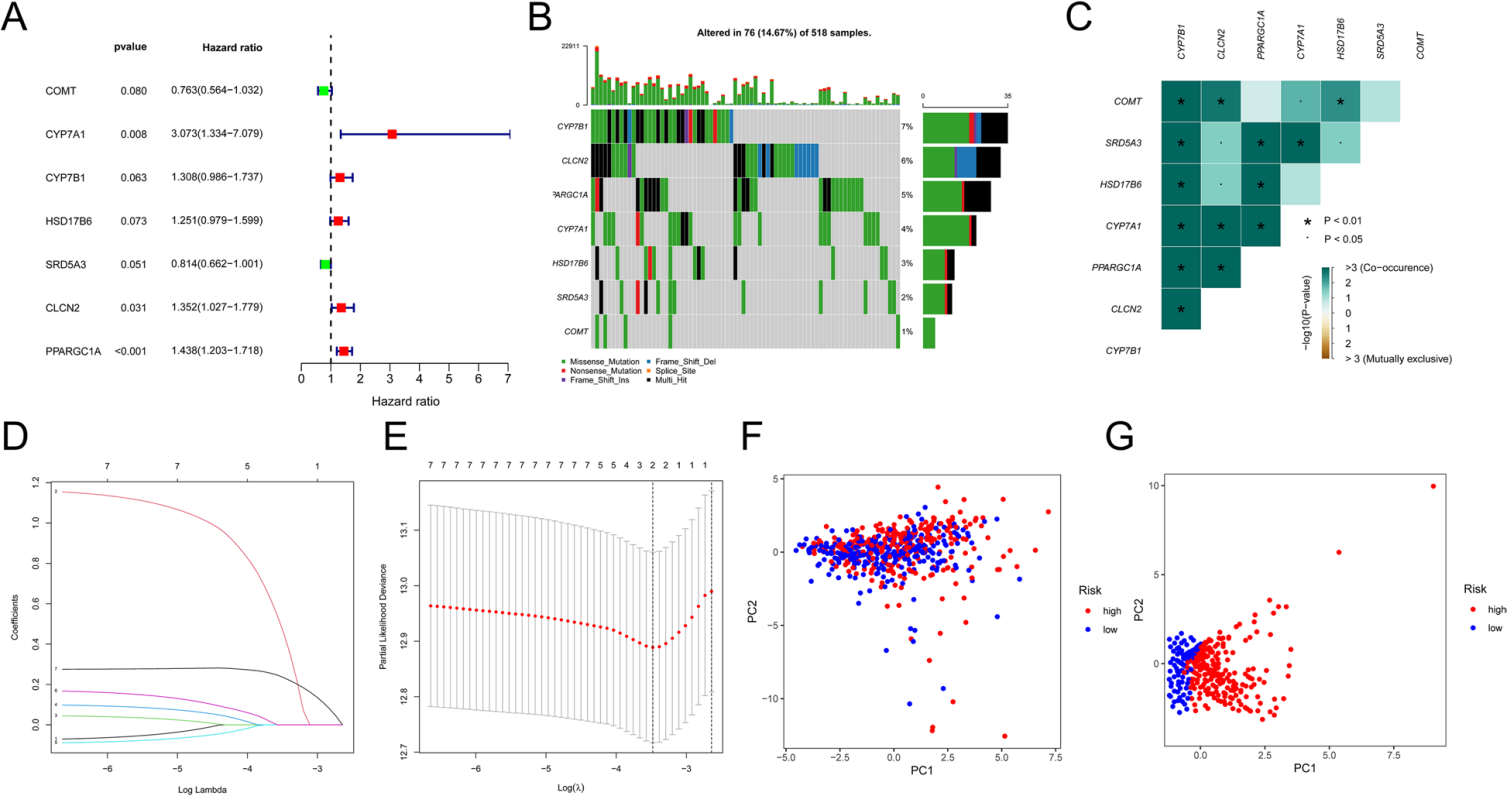
Creation of a predictive risk score model. Results of the univariate Cox regression analysis of seven genes involved in the synthesis of steroid hormones are shown in **A**. **B** The frequency of mutations in 7 genes essential for the biosynthesis of steroid hormones in 518 EC patients from the TCGA cohort. **C** Analysis of mutation co-occurrence and selection for seven genes involved in the synthesis of steroid hormones. Co-occurrence is shown by the color green, whereas exclusion is indicated by the color brown. **D** The seven genes that contribute to the synthesis of steroid hormones’ LASSO coefficients. **E** Gene identification for the development of a predictive risk score model. A line represents a gene, and the end of these genes points to a vertical axis, which is the coefficient. **F** Principal component analysis of genes involved in the steroid hormone production pathway in EC. **G** Using the model genes (CYP7A1 and PPARGC1A) of steroid hormone biosynthesis pathway, principal component analysis was performed on the TCGA cohort to separate malignant tumors from control samples. The red group represented patients at high risk, while the blue group represented patients at low risk. *P < 0.01, •*P* < 0.05

A prognostic model was constructed using EC patients from the TCGA database. Univariate Cox regression analysis was carried out, and seven genes related to steroid hormone biosynthesis were found to be associated with prognosis (*p* < 0.1) (Fig. 5A). In addition, mutations in the steroid hormone biosynthesis pathway were found in 76 out of 518 EC samples, with a frequency of 14.67%. CYP7B1 was the most frequently mutated gene, as shown in Fig. 5B.

Further analysis revealed a correlation between the coexistence of mutations in CYP7B1 and CLCN2 (as illustrated in Fig. 5C). Using LASSO Cox regression analysis, the specific gene was narrowed down. Finally, an independent prognostic score model was developed using two genes (as shown in Fig. 5D and E, and Supplement file 2). Principal component analysis demonstrated clear differentiation between the UCEC-TCGA samples (as depicted in Fig. 5F and G).

### The connection between clinical information and risk model

**Fig. 6:**
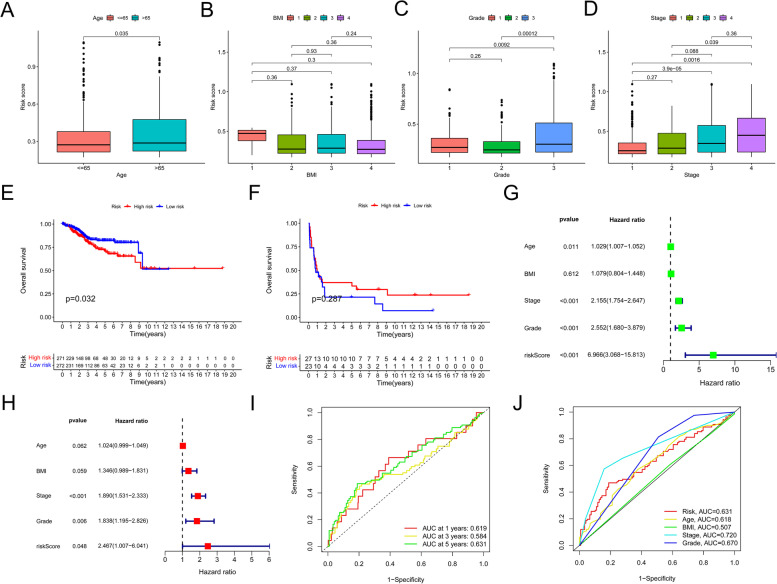
TCGA training and testing cohorts were used to construct and validate the risk model. Age (**A**), BMI (**B**), grade (**C**), and stage (**D**) are clinicopathological characteristics that are correlated with risk score (**D**). The OS curves of the subgroups were displayed using queues cohorts from TCGA (**E**) and GEO (**F**). **G**, **H** Cox regression analysis using univariate and multivariate forest plots of training cohorts. Risk score prediction of 1-, 3-, and 5-year OS in TCGA cohort. **I** ROC curve study. **J** To assess the predictive value of clinical features, ROC curves are utilized

To investigate the correlation between clinical characteristics and the predicted model, we analyzed the risk values of relevant samples based on age, BMI, grade, and stage. According to the standard range of the World Health Organization (WHO), we define all values with BMI less than 18.5 as 1, values ranging from 18.5 to 24.9 as 2, values greater than or equal to 25 as 3, and values greater than or equal to 30 as 4. Grade refers to the degree of similarity between the tumor and the originating tissue. And stage represents the range of tumor growth and dissemination. Although there was no significant difference in risk score based on BMI, age, grade, and stage showed significant differences in risk score (*p* < 0.05; Fig. 6A–D). The survival curve in Fig. 6E was analyzed using TCGA data, which includes a total of 543 samples. The survival curve in Fig. 6F was analyzed using GEO data, which includes 50 samples. Depending on the median value, the TCGA cohort was split into two risk categories, and K-M curves showed that the high-risk category had a poorer prognosis than the low-risk group (*p* < 0.05). To confirm the stability of this model, we evaluated its predictive ability in the GEO set, and the maximum AUC value for a 1-year OS was 0.619 (Fig. 6I). By displaying a time-dependent ROC at five years, the predictive risk model’s accuracy was verified (Fig. 6J). Age, grade, stage, and risk value were characteristics that predicted OS in the training and testing cohorts, as determined by univariate and multivariate analyses (Fig. 6G H).

### The creation of a survival prediction nomogram

**Fig. 7:**
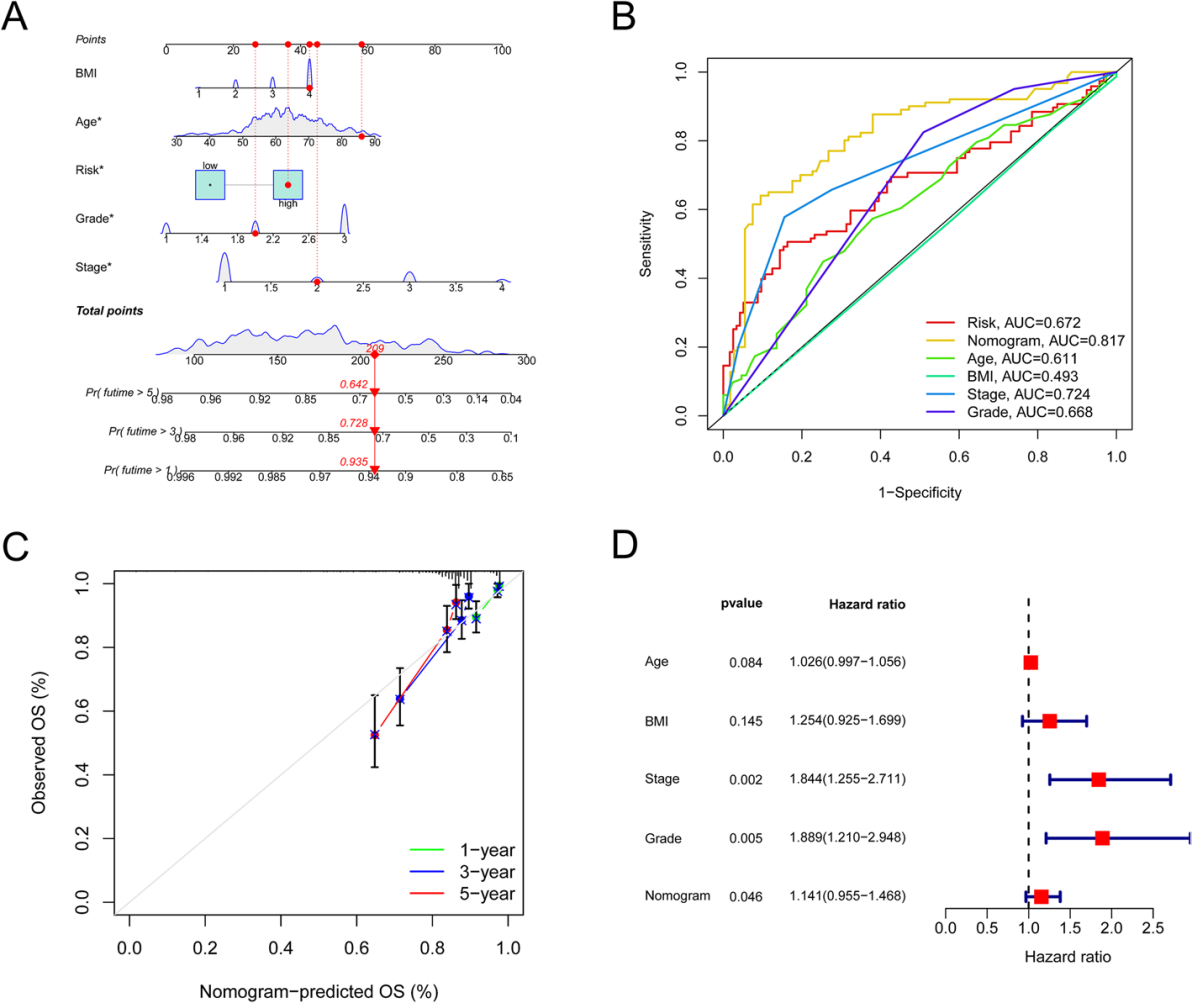
Risk score’s prognostic value in TCGA cohort OS patients when paired with clinical-pathological characteristics. **A **Nomogram in the TCGA cohort of patients reveals OS. **B** Receiver operating characteristic diagrams for the clinical and risk score components. **C** The calibration plots of the nomogram. The nomogram’s anticipated survival is shown on the x-axis, while actual survival is shown on the y-axis. **D** The Cox regression analysis of the nomogram

An OS prediction nomogram for EC tissues was developed, consisting of BMI, age, risk score, grade, and stage (Fig. 7A). The nomogram exhibited a superior predictive value (AUC = 0.817) compared to individual indicators such as grade (AUC = 0.668), stage (AUC = 0.724), or the predictive risk scoring model (AUC = 0.672; Fig. 7B). Calibration curves for 1, 3, and 5-year OS accurately predicted EC patient outcomes, as demonstrated in Fig. 7C. Cox regression analysis revealed that the predicted risk score model, grade, and stage were independent prognostic indicators (Fig. 7D).

### Immune System characteristics in high- and low-risk subgroups and response of two groups to 5-Fluorouracil

**Fig. 8:**
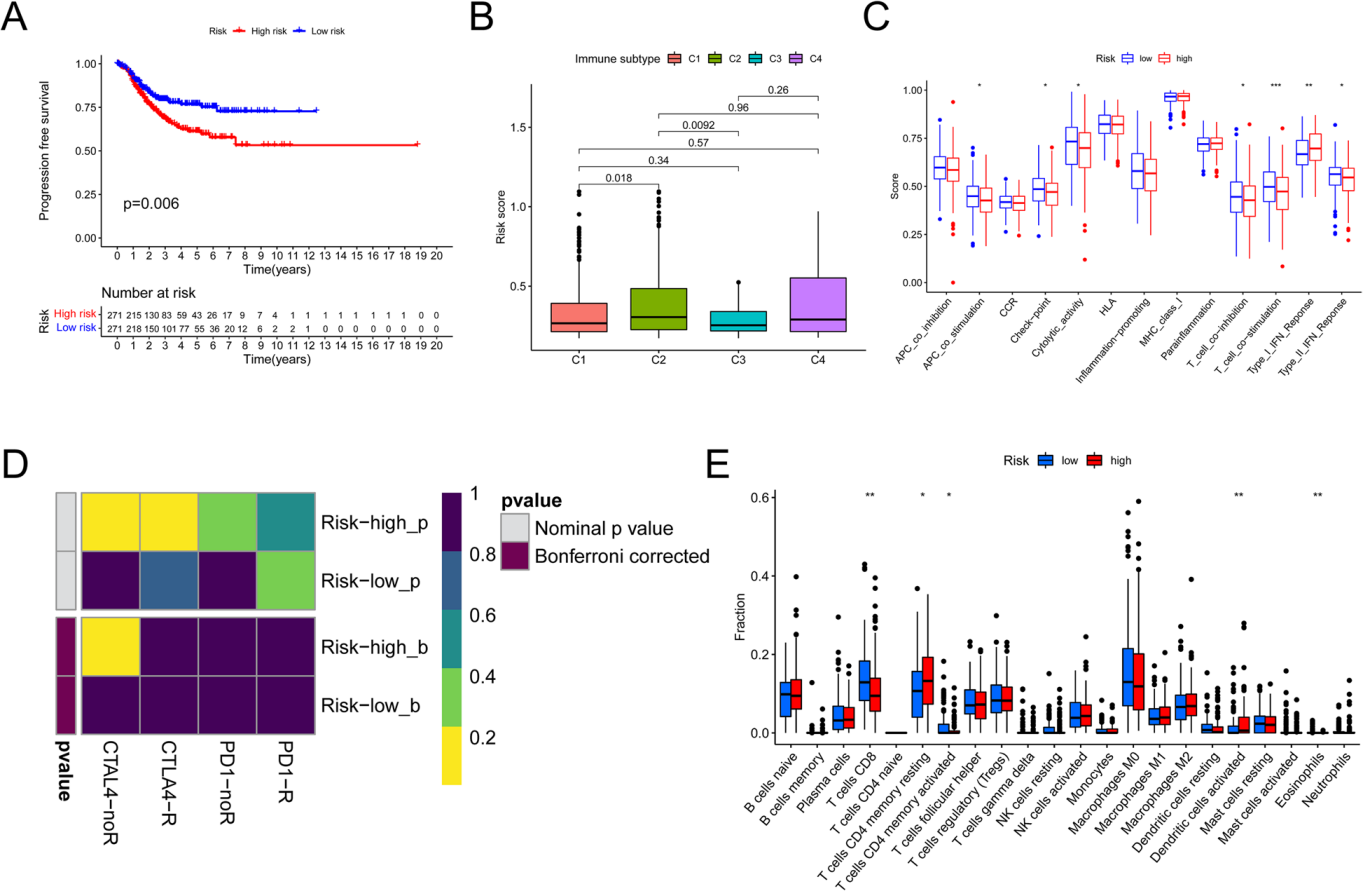
Model for predicting risk in immunotherapy. **A** The progression-free survival (PFS) rates for low- and high-risk score subgroups in the TCGA cohort were contrasted. **B** A table depicts the distribution of the immunological categories (C1, C2, C3, and C4) among risk categories. **C** The distinction between participants with high ratings and those with low ratings in terms of the immune modulation’s recognized function. **D** The effects of immunotherapy in groups at two risks. **E** The variation in immune infiltration between scores with two risks. *P < 0.05, **P < 0.01, ***P < 0.001

Significant differences in progression-free survival (PFS) were observed between the subgroups in TCGA sequencing (Fig. 8A). Immunotyping was also performed on TCGA samples, revealing significant differences in C1, C2, C3, and C4 in the high/low score categories (Fig. 8B). Additionally, there were substantial differences in type II interferon response, checkpoint, T cell co-inhibition, and T cell co-stimulation between the two groups (Fig. 8C). Increases in CD8 T cells and decreases in T cells with memory-activated CD4 were seen in the low-risk group (Fig. 8E). Immunotherapy has dramatically improved cancer treatment, for instance through CTLA4 and PD-1 inhibition. Hence, we investigated how prognostic risk score models could identify individuals who responded differently to immunosuppressive drugs. In the low and high categories, the data revealed a substantial variation in the immunological response to CTAL4 (Fig. 8D).

**Fig. 9:**
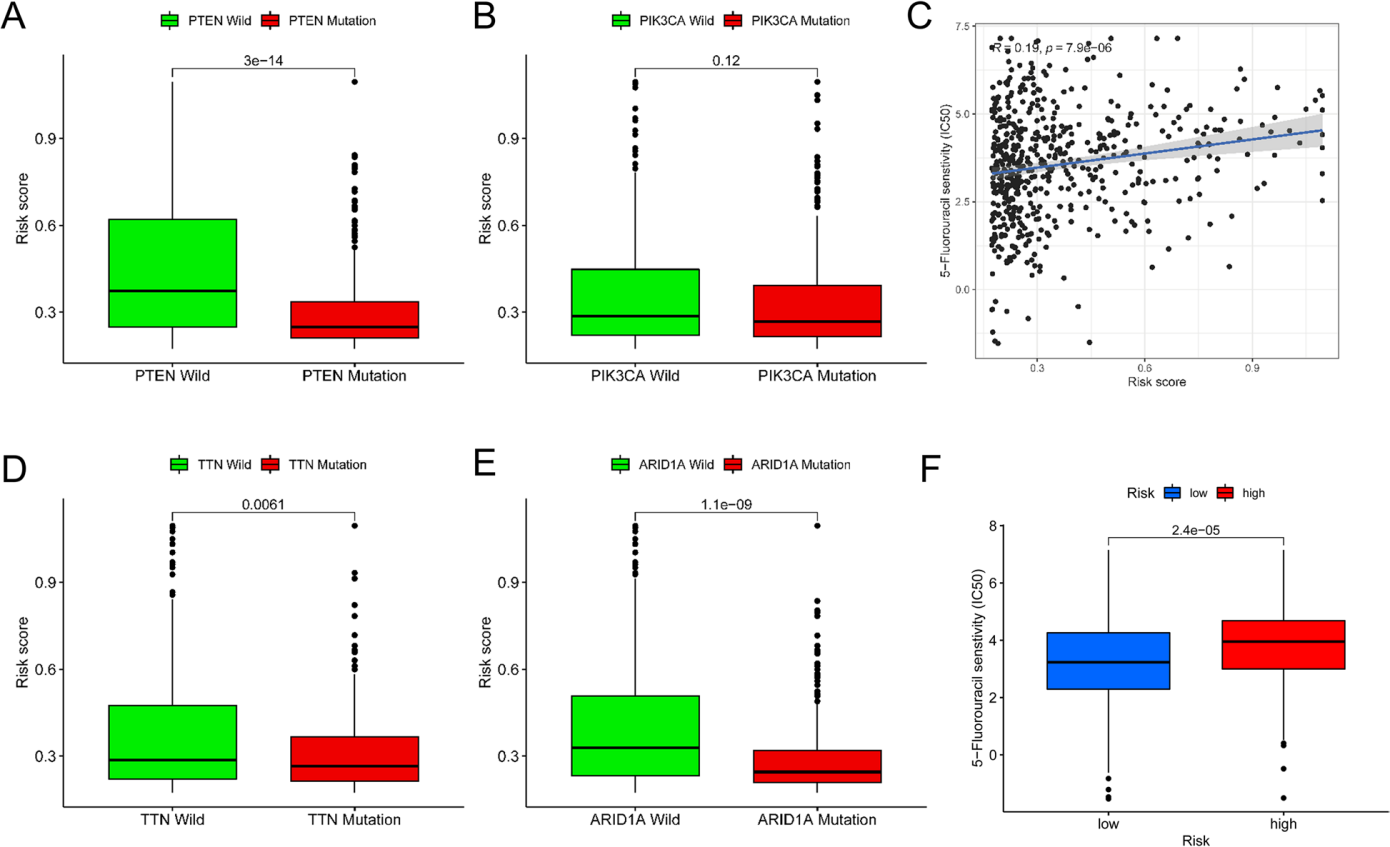
Chemotherapy-related risk prediction model. **E**, **A**, **B**, and **D** Several mutant genes have various risk ratings, including PTEN mutation (**A**), PIK3CA mutation (**B**), TTN mutation **(D**), and ARID1A mutation (**E**). **C** The correlation between the estimated IC50 value for 5-Fluorouracil and patient risk assessments. **F** Differences in the 5-Fluorouracil reaction between groups with excellent and low risk ratings

In our analysis of mutant genes with a high mutation frequency in UCEC, we found that PTEN, TTN, and ARID1A mutations were significantly associated with reduced risk ratings (Fig. 9A, B, D, and E). We then investigated the relationship between treatment resistance and risk level, as a high risk score is associated with poor outcomes. To do this, we utilized the “pRRophic” R package to determine the treatment efficacy of 5-Fluorouracil in the TCGA cohort using the half maximal inhibitory concentration (IC50). We found that samples with a lower IC50 value were more sensitive to 5-Fluorouracil treatment for UCEC (Fig. 9C, F).

### Hub gene validation of the prediction model in DEGs of both two categories

**Fig. 10:**
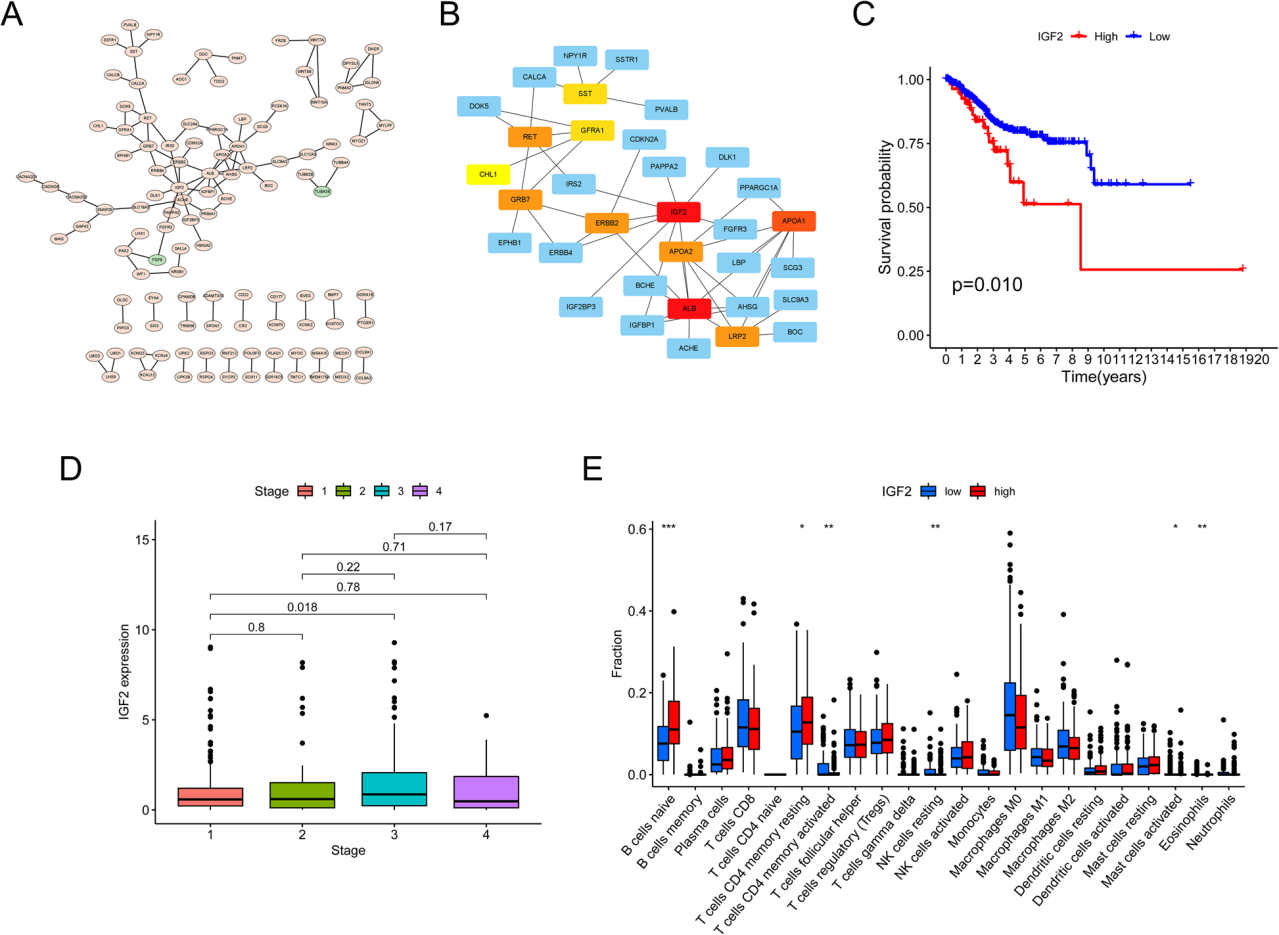
PPI graph for protein-protein interactions. **A** Cytoscape-generated PPI network (red) depicts DEGs that demonstrated strongly in the good score category; green depicts DEGs that demonstrated strongly in the low-risk score category. **B** CytoHubba selected the top eleven hub genes. **C** Analysis of patient mortality broken down into subgroups according to IGF2 mRNA expression. **D** The variation in IGF2 mRNA expression throughout different phases. **E** The quantity of TME-infiltrating cells was higher/lower in participants with elevated/low IGF2 mRNA expression. *p < 0.05; **p < 0.01

The interactions between differentially expressed genes (DEGs) were analyzed and visualized using Cytoscape. Up-regulated genes in the high-risk subgroup were represented in red, while up-regulated genes in the low-risk subgroup were in green (Fig. 10A). The Cytohubba Cytoscape plug-in was utilized to identify the central gene among the DEGs, and 11 genes were selected (Fig. 10B). IGF2 was then confirmed as the hub gene. Survival analysis showed a strong correlation between IGF2 expression levels and mortality in EC patients (Fig. 10C). Furthermore, IGF2 expression was found to increase in stage 3 and decrease in stage 4 (Fig. 10D). We also investigated the association between IGF2 expression levels and immune infiltration in the tumor microenvironment (TME). Patients with low IGF2 expression had significantly lower levels of B cells and resting memory CD4 + T cells in their tumors compared to those with high IGF2 expression (Fig. 10E).

### IGF2 promoted EC cell proliferation and migration

**Fig. 11:**
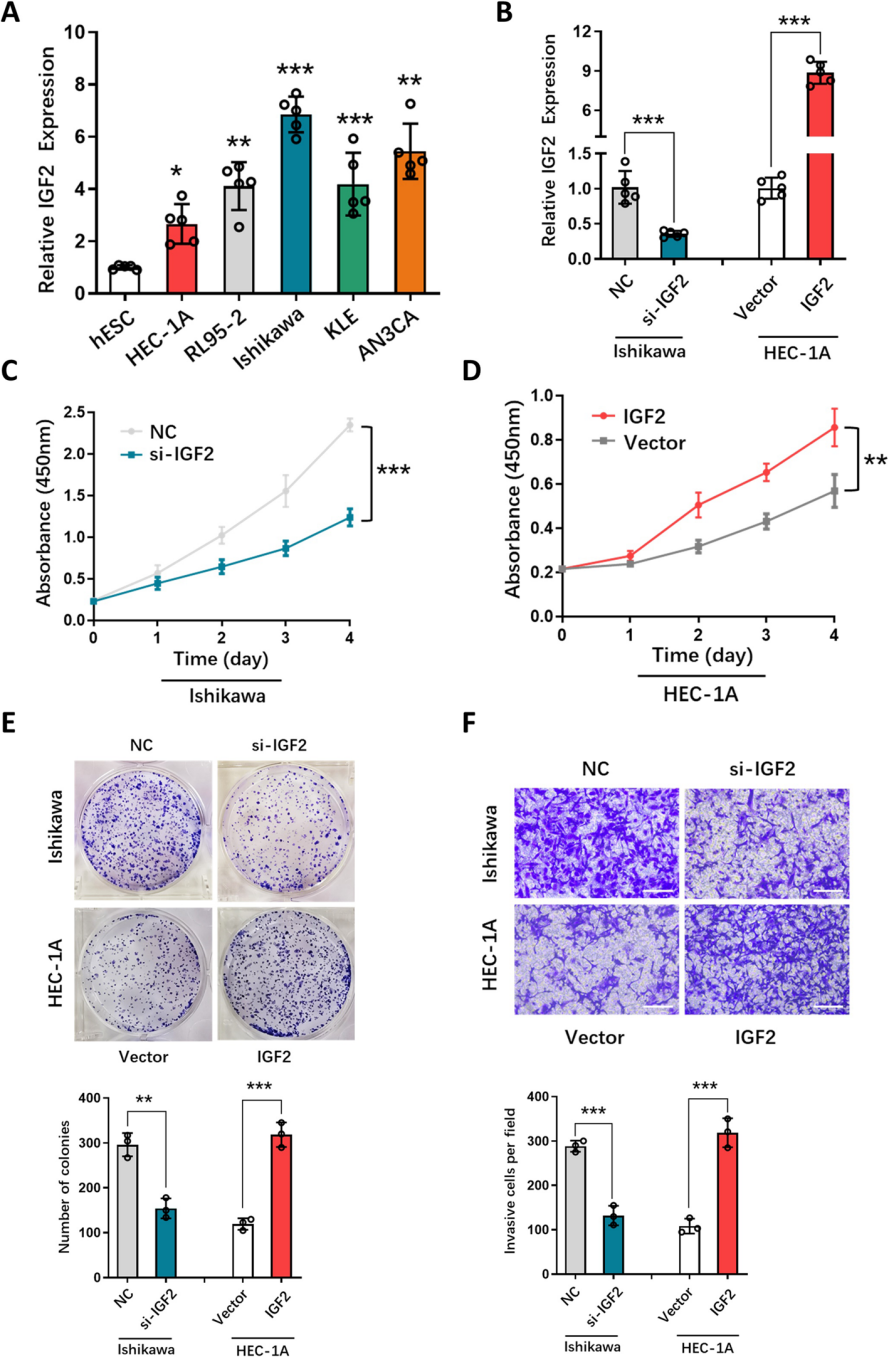
Proliferation and Migration of EC Cells were Encouraged by IGF2. IGF2 mRNA expression was detected by qRT-PCR in hESC and EC cells (**A**). In Ishikawa and HEC-1 A cells transfected with si-IGF2 and IGF2-cDNA, IGF2 levels were measured by qRT-PCR (**B**). In transfected Ishikawa and HEC-1 A cells, CCK-8 represents the cell growth curve. Colony test on transfected Ishikawa and HEC-1 A cells (**E**). Transfected Ishikawa and HEC-1 A cells were tested for transwell invasion (**F**). *P < 0.05,**P < 0.01, ***P < 0.001

To further validate the role of IGF2 in EC, we investigated whether changes in its expression affected EC cell proliferation and malignancy. Initially, we measured IGF2 expression levels in various EC cell lines and found that HEC-1 A, RL95-2, Ishikawa, KLE, and AN3CA cells had significantly higher levels of IGF2 mRNA than hESCs (Fig. 11A). We chose Ishikawa cells for si-IGF2 transfection and HEC-1 A cells for IGF2-cDNA transfection. As a result, si-IGF2 reduced IGF2 expression in Ishikawa cells, while IGF2-cDNA increased IGF2 expression in HEC-1 A cells (Fig. 11B). CCK-8 and colony formation assays demonstrated that upregulating IGF2 expression promoted HEC-1 A cell proliferation, while downregulating it inhibited Ishikawa cell proliferation (Fig. 11C, D, and E). Transwell assays showed that suppressing IGF2 expression decreased the invasion of Ishikawa cells, whereas upregulating it significantly increased the invasion of HEC-1 A cells (Fig. 11F). These results suggest that knocking down IGF2 inhibits EC cells’ growth and migration.

## Discussion

The coexistence of PCOS and EC has been previously documented, but the exact underlying pathophysiology of PCOS remains unclear [9]. As previously mentioned, PCOS patients have an increased risk of EC, with obesity and insulin resistance identified as contributing factors [8]. Although the relationship between obesity and insulin resistance with increased risk of EC in PCOS patients is well-established, there is limited research on the genetic characteristics of EC in PCOS. In this study, we utilized the WGCNA algorithm to identify common processes between PCOS and EC for the first time. By specifically examining genes associated with clinical characteristics and co-expression modules, we were able to gain significant biological insights into the shared pathophysiology of these conditions [12].

The biosynthesis of steroid hormones has been identified as a crucial feature in both PCOS and EC. As the human endometrium is subject to the regulation of steroid hormones, which govern uterine growth, the overstimulation of endometrial epithelial cells by estrogen in comparison to progesterone is frequently cited as the principal mechanism underlying the development of endometrial cancer [17]. The clinical feature of PCOS is an imbalance of sex steroid hormones, which results in the overstimulation of endometrial epithelial cells by estrogen. When progesterone fails to balance estrogen, the endometrium begins to grow, which may lead to the development of cancer cell clones due to oncogenes and tumor suppressor genes being more susceptible to random mutations. Without proper repair mechanisms, endometrial cells with numerous mutations may gain a growth advantage [18]. Various studies have shown that the growth of tumors can be stimulated by estrogen, which induces the production of aromatase in stromal cells within the tumor. This increase in aromatase can cause the tumor cells to produce estrogen locally, leading to further tumor growth [19]. Studies have indicated that the expression and function of aromatase may contribute to the poor prognosis and aggressiveness of endometrial cancer [20].

In contrast, 17-hydroxysteroid dehydrogenase (17-HSD-1) is upregulated in EC, leading to an increased production of active 17-estradiol (E2) from low active estrone (E1) within the tissue. This suggests a higher level of intra-tissue E2 production in this estrogen-dependent condition [21]. In an independent study, mice treated with E1 and 17-HSD-1 inhibitors exhibited a marked reduction in tumor growth of approximately 65% compared to those treated with E1 alone [22]. While most current studies focus on individual genes, the collective impact of all genes involved in steroid hormone synthesis on EC remains unclear. Therefore, comprehending the involvement of steroid hormone production in tumorigenesis and its interaction with immunotherapy may facilitate the identification of optimal treatment strategies.

Numerous prognostic models for EC that incorporate immunological or methylation-related elements have been effectively constructed [23–25]. In this work, we looked at the relationship between PCOS and EC in the production of steroid hormones. Our analysis based on GO and KEGG databases identified 78 genes closely associated with the process. We further developed a scoring model utilizing prognosis-related genes from the 78 genes. In addition, we evaluated the performance of the predictive model in a GEO cohort and investigated the clinical significance of the identified genes in UCEC through correlation analysis. The survival rates between the low-risk and high-risk subgroups showed a clear difference. The ROC curve was used to further evaluate the prediction model’s accuracy.

Although surgery is typically effective in treating early-stage endometrial cancer, advanced cases can be challenging, with poor prognoses, especially in cases of metastasis or recurrence [26]. Chemotherapy is a key component in the adjuvant treatment of endometrial cancer, particularly for patients with advanced disease, as it can reduce the risk of recurrence, mitigate distant metastases, and improve overall survival rates [27–29]. To gain more insight into the applicability of the predictive risk model for EC, we investigated the response of patients in the two risk categories to pharmaceutical therapy. Specifically, we examined the response to the traditional antimetabolic anticancer drug 5-fluorouracil [30]. In addition, 5-fluorouracil has been reported to be effective in reversing carboplatin resistance in various types of cancer. Bruckner et al. conducted experiments on endometrial cancer and demonstrated the activity and synergistic effects of 5-fluorouracil [31]. Previous studies have found a positive association between risk score and resistance to 5-Fluorouracil [32]. The customized risk score model we developed can offer valuable insights into the prognosis of EC patients. Chemotherapy is often avoided in high-risk patients due to the likelihood of developing chemotherapeutic resistance. Immunotherapy, which involves blocking immunological checkpoints such as PD-1/L1 and CTLA4, is generally not recommended for patients with poor EC prognosis. Therefore, clinical practice should identify patients who are eligible for immunotherapy. Patients with low scores exhibit a high level of immunological inflammatory and inhibitory cells. Additionally, low-scoring patients may trigger an IFN response that exacerbates inflammation. These findings support the prediction of immune dysfunction and resistance in cancer, suggesting that patients with low scores may be suitable candidates for immunotherapy.

After analyzing the distinct genes in the two groups, we proceeded to investigate the hub gene, IGF2, which emerged as a significant candidate from the PPI network. IGF2, a polypeptide involved in cell proliferation and growth, is a critical component of the insulin/IGF-signaling pathway that regulates metabolic functions [33]. IGF2 is crucial for carcinogenesis and aids in the development of cancer [34]. IGF-2 has been observed to be activated and expressed inappropriately in several malignancies, including but not limited to hepatocellular carcinoma, colon cancer, liposarcoma, and embryonic tumors [35–38]. Continuous IGF2 activity raises the possibility of transformation. Animals that overexpress IGF2 are at an increased likelihood of developing mammary gland adenocarcinoma [39] as well as lung cancer [40]. The association between increased IGF2 expression and poorer prognosis is well established, as shown by the elevated mortality in breast cancer [41], shorter recurrence times in oesophageal cancer [42], and faster progression in chronic myeloid leukemia [43]. Peters et al [44]. IGF2 promotes the production of endothelial growth factor (VEGF) and interacts with it to encourage vasculogenesis. Inhibition of IGF2 expression decreased VEGF levels and slowed development [45]. Furthermore, IGF2 increases steroid synthesis, contributing to prostate cancer progression [46]. Despite its importance, little is known about IGF2’s role in carcinoma. Our findings are consistent with past studies in that IGF2 mRNA expression was linked to a worse prognosis and clinical stage. According to Jasminka et al., the levels of IGF1R and IGF2 in advanced-stage (scenes III–IV) malignant tissues were significantly greater than in early stages or endometrial hyperplasia, indicating that overexpression of the IGF2 gene is linked to a bad prognosis in endometrial cancer [47]. Furthermore, several in vivo investigations have shown that neutralizing antibodies may effectively target the IGF1R and INSR-A signal transduction pathways while inhibiting IGF1 and IGF2 ligands, which have a powerful anticancer impact [48]. So far, IGF2 has not been adequately studied in endometrial cancer, and more attention needs to be paid to its role in endometrial cancer for better therapeutic interventions.

## Conclution

This study has identified the biosynthesis of steroid hormones as a potential mechanism for PCOS patients’ susceptibility to EC. Through the development of a predictive scoring model according to pathway-related genes, the influence of these genes on EC prognosis has been demonstrated. Additionally, a connection between the steroid hormone biosynthesis pathway and the immunological characteristics of EC has been established, providing potential opportunities for tailored cancer immunotherapy in the future.

## Supplementary Information

**Additional file 1:** **Table 1. **Genes related to the steroid hormone biologicalprocess.

**Additional file 2: Table 2.** Two genes were used to build an independent prognosticscore model.

## Data Availability

The datasets used during the present study are available from GEO (https://www.ncbi.nlm.nih.gov/geo/) database and the TCGA database (https://portal.gdc.cancer.gov/).
